# PrP^C^ from stem cells to cancer

**DOI:** 10.3389/fcell.2014.00055

**Published:** 2014-09-29

**Authors:** Séverine Martin-Lannerée, Théo Z. Hirsch, Julia Hernandez-Rapp, Sophie Halliez, Jean-Luc Vilotte, Jean-Marie Launay, Sophie Mouillet-Richard

**Affiliations:** ^1^Toxicology, Pharmacology and Cellular Signaling, INSERM UMR-S1124Paris, France; ^2^Toxicology, Pharmacology and Cellular Signaling, Université Paris Descartes, Sorbonne Paris Cité, UMR-S1124Paris, France; ^3^Université Paris Sud 11, ED419 BiosigneOrsay, France; ^4^U892 Virologie et Immunologie Moléculaires, INRAJouy-en-Josas, France; ^5^UMR1313 Génétique Animale et Biologie Intégrative, INRAJouy-en-Josas, France; ^6^AP-HP Service de Biochimie, Fondation FondaMental, INSERM U942 Hôpital LariboisièreParis, France; ^7^Pharma Research Department, F. Hoffmann-La-Roche Ltd.Basel, Switzerland

**Keywords:** cellular prion protein, stem cell, cancer, self-renewal, cell fate specification, prion infection

## Abstract

The cellular prion protein PrP^C^ was initially discovered as the normal counterpart of the pathological scrapie prion protein PrP^Sc^, the main component of the infectious agent of Transmissible Spongiform Encephalopathies. While clues as to the physiological function of this ubiquitous protein were greatly anticipated from the development of knockout animals, PrP-null mice turned out to be viable and to develop without major phenotypic abnormalities. Notwithstanding, the discovery that hematopoietic stem cells from PrP-null mice have impaired long-term repopulating potential has set the stage for investigating into the role of PrP^C^ in stem cell biology. A wealth of data have now exemplified that PrP^C^ is expressed in distinct types of stem cells and regulates their self-renewal as well as their differentiation potential. A role for PrP^C^ in the fate restriction of embryonic stem cells has further been proposed. Paralleling these observations, an overexpression of PrP^C^ has been documented in various types of tumors. In line with the contribution of PrP^C^ to stemness and to the proliferation of cancer cells, PrP^C^ was recently found to be enriched in subpopulations of tumor-initiating cells. In the present review, we summarize the current knowledge of the role played by PrP^C^ in stem cell biology and discuss how the subversion of its function may contribute to cancer progression.

## Introduction

The discovery of the cellular prion protein PrP^C^ dates back to 1985 with the identification that the scrapie prion protein PrP^Sc^, the main component of the infectious agent responsible for Transmissible Spongiform Encephalopathies (TSEs) was encoded by a gene of the host, termed *Prnp* (Oesch et al., 1985). PrP^C^ has been extensively scrutinized as the endogenous substrate for conversion into its pathogenic PrP^Sc^ counterpart (Aguzzi and Calella, 2009), while studies on its physiological function have long been overlooked. At the molecular and cellular levels, it is well established that PrP^C^ is anchored to the outer leaflet of the plasma membrane through a glycosyl-phosphatidylinositol (GPI) moiety (Linden et al., 2008). It may exist under a great diversity of isoforms as a result of heterogeneous glycosylation (Ermonval et al., 2003) and proteolytic cleavage (McDonald et al., 2014). Although it is suspected that the wide repertoire of PrP^C^ species may endow the protein with the capacity to interact with multiple soluble ligands, extracellular matrix components or cell-surface proteins, the specific tissue distribution, and function of each isoform remain elusive (Linden et al., 2008). That research on PrP^C^ function has lagged behind that of TSE pathophysiology may notably be explained by the lack of major abnormalities in PrP-null mice (Steele et al., 2007), whose most obvious phenotype is their resistance to TSE agents (Bueler et al., 1993). Because PrP is ubiquitously expressed and very much conserved in mammals, with *Prnp* orthologs identified in fish, birds, and reptiles (Premzl and Gamulin, 2007), the apparent normal phenotype of PrP null mice was quite unexpected and proposed to reflect the occurrence of compensatory mechanisms. One major contribution of these mice, however, was the demonstration that PrP^C^ is mandatory for the long-term repopulating activity of hematopoietic stem cells (HSCs) (see below) (Zhang et al., 2006). This seminal report set the stage for investigating into the role exerted by PrP^C^ in stem cell biology. Here, we provide an overview of the recent advances regarding the contribution of PrP^C^ to stem cell biology and their pathophysiological implications.

## PrP^C^ expression and role during development

Studies on PrP^C^ have initially focused on the adult central nervous system (CNS), since it is the only target of PrP^Sc^-associated toxicity (Aguzzi and Calella, 2009). Further, PrP^C^ is most abundantly found in neurons (Linden et al., 2008). Notwithstanding, PrP^C^ is highly expressed during embryonic development, as first shown by Manson et al. over two decades ago (Manson et al., 1992). This *in situ* hybridization analysis revealed widespread expression of *Prnp* transcripts in the developing central and peripheral nervous system at embryonic days E13.5 and E16.5, as well as in other tissues such as the intestine or the dental lamina (Manson et al., 1992). *Prnp* mRNA was also detected in extra-embryonic tissues from E6.5, pointing for the first time to a potential role for PrP^C^ in the placenta, which has started to be accurately assessed recently (Alfaidy et al., 2012; Passet et al., 2012). These first data were refined with the detection of *Prnp* mRNA starting at E8.5–E9 in the differentiating neuroepithelium (Miele et al., 2003). The induction of *Prnp* expression at this stage in the developing CNS and heart was confirmed in a study using *Prnp*-LacZ reporter mice (Tremblay et al., 2007).

Based on this developmental pattern of expression, transcriptomic analyses were carried out on early *Prnp* knockout vs. wild-type (WT) embryos and revealed prominent alterations, with a total number of 263 genes differentially expressed at day E7.5 (Khalife et al., 2011). The array of genes with altered expression notably includes a set of growth factors and growth factor receptors, supporting the notion that PrP^C^ plays an important role in the regulation of cascades associated with embryonic development (Khalife et al., 2011). Interestingly, the pattern of pathways affected overlaps with that obtained after early embryonic gene expression profiling of zebrafish PrP2 morphants (Nourizadeh-Lillabadi et al., 2010).

The zebrafish model actually allowed bringing to light a vital function for PrP^C^ since morpholino-mediated knockdown of the PrP ortholog PrP1 in this species leads to loss of embryonic cell adhesion and gastrulation arrest (Malaga-Trillo et al., 2009). Of note, the defects observed could be rescued with mouse *Prnp* mRNA, indicating that this function is evolutionary conserved (Malaga-Trillo et al., 2009). Thus, the overall data gained at the animal scale argue that PrP^C^ fulfills an important function during embryogenesis and that its ablation in mice triggers the implementation of yet-to-be-deciphered compensatory mechanisms.

## PrP^C^ regulates the self-renewal of stem/progenitor cells

The link between PrP^C^ and stem cell biology was first uncovered in HSCs. Investigations of PrP^C^ in the hematopoietic system were initially prompted by the observation that PrP^Sc^ accumulates in lymphoid organs and by the quest to understand the cellular mechanisms sustaining prion propagation in the periphery (Mabbott and MacPherson, 2006). These studies demonstrated that PrP^C^ is highly expressed at the surface of various hematopoietic cells, including B and T lymphocytes, monocytes, dendritic cells, megakaryocytes and platelets, but not erythrocytes or granulocytes (Linden et al., 2008). In the human bone marrow, PrP^C^ was found to be present in the CD34^+^ stem / progenitor cell population (Dodelet and Cashman, 1998) and to be preferentially expressed on murine CD43^+^, B220^−^, IL-7R^−^ cells, enriched in immature progenitors (Liu et al., 2001). A major advance was the discovery by the team of Lodish that PrP^C^ is very abundant at the surface of mouse bone marrow Lin^−^Sca^+^Endoglin^+^ cells, a population comprising immature HSCs (Zhang et al., 2006). These authors then assessed the ability of bone marrow-derived Lin^−^Sca^+^Endoglin^+^ cells from *Prnp* knockout mice to reconstitute the hematopoietic system of lethally irradiated mice in serial transplantation assays, and demonstrated that HSCs from *Prnp* null mice lack long-term repopulating activity (see Table 1) (Zhang et al., 2006). These experiments further allowed substantiating that the Lin^−^Sca^+^Endoglin^+^ cell population endowed with long-term repopulating activity is PrP^C^ positive (Zhang et al., 2006).

**Table 1:** **Summary of effects of PrP^C^ depletion on stem and progenitor cells**.

**Cell type/tissue**	**PrP^C^-null/KD vs. wt**	**References**
**EMBRYO/POSTNATAL**		
ES	Increased apoptosis in embryoid bodies	Miranda et al., 2011
Human ES	Inhibition of ectodermal differentiation	Lee and Baskakov, 2012
	Reduced transition from G1 to S phase during spontaneous differentiation	
Multipotent neural precursors E13.5 (telencephalon)	Delayed neuronal differentiation	Steele et al., 2006
Neurospheres isolated from E14 forebrain	Reduced neurosphere formation	Santos et al., 2011
E16.5 oligodendrocyte precursor cells (OPC) in optic nerve explants	Increased proliferation (BrdU incorporation)	Bribian et al., 2012
E16.5 neocortex	Increased NG2^+^ Olig2^+^ OPC	Bribian et al., 2012
P0-P2 cortical OPC primary culture	Delayed differentiation	Bribian et al., 2012
P5 neurospheres isolated from SVZ	Reduced proliferation (BrdU incorporation) and secondary neurosphere formation	Prodromidou et al., 2014
**ADULT**		
Dentate gyrus	Reduced proliferation (BrdU incorporation)	Steele et al., 2006
	Reduced proliferation of neuroprogenitors and/or neurogenesis (BrdU^+^ NeuN^+^)	Bribian et al., 2012
Neocortex	Increased NG2^+^ Olig2^+^ OPC	Bribian et al., 2012
SVZ	Reduction of cycling GFAP^+^ neural stem cells	Prodromidou et al., 2014
	Decreased Mash^+^ progenitors and accumulation of proliferating neuroblasts DCX^+^	
HSC	Loss of long-term repopulating activity in serial transplantation assays	Zhang et al., 2006
Skeletal muscle	Delayed regeneration after injury (retarded exit from the cell cycle of myogenic precursor cells)	Stella et al., 2010
Human MSC	Reduced clonogenic potential, proliferation and differentiation	Mohanty et al., 2012

Much like HSCs, neuroepithelial stem cells have the capacity to proliferate through repeated symmetric divisions and generate radial glial cells, which then undergo asymmetric division and give rise to neurons, oligodendrocytes, and astrocytes (Gotz and Huttner, 2005). The proper proceeding of these expansive and neurogenic phases is crucial for the development of the CNS. By comparing neural stem cells isolated from *Prnp* knockout, WT or PrP overexpressing mice at embryonic day 13.5, Steele et al. documented that PrP^C^ levels directly increase the differentiation rate of multipotent neural precursor cells (Steele et al., 2006). In the same study, PrP^C^ expression levels were further found to correlate with the proliferation rate in the two adult neurogenic regions, the subventricular zone (SVZ) or the dentate gyrus (DG) (Steele et al., 2006). The latter finding was corroborated by two independent studies showing that the formation of neurospheres from fetal (Santos et al., 2011) or postnatal (Prodromidou et al., 2014) brains is less efficient with *Prnp* knockout than WT mice.

The notion that PrP^C^ contributes to the proliferation of stem cells extends beyond hematopoietic and neural stem cells. Indeed, the level of PrP^C^ was found to serve as an effective cell surface marker for self-renewing mammary gland stem cells in mice (Liao et al., 2007). More recently, PrP^C^ was further shown to promote the expansion and engraftment of bone marrow-derived human mesenchymal stem cells (MSCs) (Mohanty et al., 2012). Finally, PrP^C^ was shown to exert either an anti- or a pro-proliferative effect in human embryonic stem (ES) cells, depending on whether they are grown under self-renewing or differentiating conditions, respectively (Lee and Baskakov, 2012).

## PrP^C^ influences stem cell fate

The identification of PrP^C^ as a broad cell surface marker for stem / progenitor cells raises the question as to whether the expression of PrP^C^ is a determinant of the stem cell fate. In this respect, we (Mouillet-Richard et al., 1999) and others (Peralta et al., 2011) have provided evidence that PrP^C^ is upregulated following the cell fate restriction of multipotential ES or embryonic carcinoma (EC) cells toward the neuronal lineage. Similarly, PrP^C^ is induced in ES-derived cardiomyogenic progenitors obtained after embryoid body (EB) formation (Hidaka et al., 2010). In line with this, the expression of PrP^C^ was found to be increased during spontaneous differentiation of mouse and human ES cells (Lee and Baskakov, 2010; Miranda et al., 2011) and, reciprocally, induction of PrP^C^ in human ES cells grown under self-renewal conditions was shown to promote their differentiation (Lee and Baskakov, 2012). Intriguingly, exposure of ES cells to recombinant PrP delays their spontaneous differentiation (Lee and Baskakov, 2010). In view of the early expression of PrP^C^ in extra-embryonic tissues, it is tempting to speculate that placenta-derived PrP^C^ may serve as a paracrine signal to maintain the self-renewal of inner mass cells, until their appropriate induction toward either of the three lineages.

Beyond lineage specification, the regulation of PrP^C^ expression also accompanies differentiation toward a given fate. Along the hematopoietic lineage, PrP^C^ appears to be downregulated upon differentiation of CD34^+^ progenitors toward a granulocytic fate, while its expression is retained in B and T lymphocytes as well as monocytes (Dodelet and Cashman, 1998). In addition, PrP^C^ is absent from erythrocytes (Dodelet and Cashman, 1998) and abundant in megakaryocytes and platelets (Starke et al., 2005), suggesting that the expression of PrP^C^ is switched off with the commitment of megakaryocytic-erythrocytic progenitors toward the erythroid fate, or is decreased along erythroid differentiation, in line with (Panigaj et al., 2011).

As for neural progenitor cells, the expression of PrP^C^ was reported to be increased along neuronal differentiation, while barely detected in astrocytes or oligodendrocytes (Steele et al., 2006). This high neuronal PrP^C^ expression is in line with the well-documented contribution of PrP^C^ to neuronal differentiation, including neurite outgrowth (Chen et al., 2003; Santuccione et al., 2005; Loubet et al., 2012; Santos et al., 2013) or synapse maturation (Kanaani et al., 2005). The lack of PrP^C^ detection in differentiating oligodendrocytes and astrocytes in the study by Steele et al. (2006) is, however, in contrast with several reports documenting an abundant PrP^C^ expression in these two cell types in late embryos or in the postnatal brain (Moser et al., 1995; Lima et al., 2007; Bribian et al., 2012). Interestingly, both oligodendrocytic (Bribian et al., 2012) and astrocytic (Arantes et al., 2009) differentiation kinetics appear to be delayed in *Prnp* knockout mice. These observations recall the delay in neuronal differentiation, as initially reported by Steele et al. (2006), as well as the slower regeneration of muscle after injury (Stella et al., 2010) in a PrP null context. Whether PrP^C^ expression affects the balance from one fate to another remains, however, to be investigated. In this regard, it is worth noting that prion infection in adult neural stem cells (NSCs) favors the differentiation toward the glial lineage at the expense of neuronal differentiation (Relaño-Ginés et al., 2013).

## Stem cells and prion replication

Whether stem cells are susceptible to prion infection may at first seem a question without relevance, since TSEs are neurodegenerative diseases. However, as rightly underlined in the study by Relaño-Ginés (Relaño-Ginés et al., 2013), exploiting the potential of adult NSCs is currently considered as a promising avenue to mitigate neurodegeneration (Bellenchi et al., 2013). While several studies had reported an efficient replication of PrP^Sc^ in neurospheres isolated from fetal brain (Milhavet et al., 2006; Herva et al., 2010), the susceptibility of adult NSC toward prion infection has been evaluated only recently. In line with the results obtained with embryonic-derived cultures, neurospheres isolated from the SVZ or the DG of adult mice were shown to support prion replication (Relaño-Ginés et al., 2013). The same study further documented the presence of dense PrP^Sc^ deposits in the DG and the SVZ of prion-infected mice, indicating that prions colonize adult NSCs, the brain's endogenous repair machinery (Relaño-Ginés et al., 2013, 2014). Of note, prion replication of adult NSCs was found to impair neuronal differentiation, both *in vitro* and *in vivo* (Relaño-Ginés et al., 2013). Thus, in addition to constituting a reservoir of PrP^Sc^, the replication of prions in adult NSCs may also compromise the regeneration of damaged neurons. Finally, because PrP^Sc^ is known to deviate the normal function of PrP^C^ (Westergard et al., 2007; Pradines et al., 2013), these observations suggest that studying the impact of prion infection on the self-renewal and fate of NSCs may improve our understanding of the physiological role exerted by PrP^C^ in these processes.

## PrP^C^-dependent control of stem cell self-renewal and fate: mechanistic insight

Notwithstanding the well-established involvement of PrP^C^ in the self-renewal of diverse types of stem /progenitor cells, the molecular mechanisms at play remain obscure. One possible mode of action of PrP^C^ would be through the interaction with one of its ligands. This view is clearly supported by the demonstration that the binding of PrP^C^ with STI-1 is critical for the formation and proliferation of neurospheres cultured from fetal forebrain (Santos et al., 2011). While several signaling cascades elicited by the interaction of STI-1 with PrP^C^ have been described in a neuronal context (Hirsch et al., 2014), the pathways mobilized to sustain neurosphere self-renewal and proliferation have not been analyzed so far (Santos et al., 2011). On another hand, the presence of PrP^C^ on neurospheres was recently shown to be required for NCAM-induced neuronal differentiation (Prodromidou et al., 2014). These two sets of observations raise the question as to the PrP^C^ isoforms that respectively bind STI-1 and NCAM, since these two molecules instruct distinct responses. Another PrP^C^ partner that may have relevance to stem cell biology is the amyloid precursor protein APP, whose functional interaction with PrP in the zebrafish modulates cell adhesion and CNS development (Kaiser et al., 2012). Whether the APP-PrP^C^ interaction is involved in the regulation of E-cadherin-dependent adhesion in zebrafish embryos deserves further investigation (Malaga-Trillo et al., 2009). Interestingly, our own studies on a neuroectodermal stem cell line also substantiate a disruption of cadherin-mediated cell contacts upon PrP^C^ depletion (Martin-Lannerée et al., unpublished observations). It is of note that cell adhesion processes are now recognized as major determinants of stem cell biology in relation with their local microenvironment (stem cell niche) (Marthiens et al., 2010). By affecting adhesion properties of stem cells, the depletion of PrP^C^ may thus in turn impact on their retention, self-renewal or exit from their niche.

## A role for PrP^C^ in cancer stem cells?

The contribution of PrP^C^ to cell proliferation appears to apply to many cell types beyond stem/progenitor cells. These notably include cancer cells, as first demonstrated in gastric tumor cell lines (Liang et al., 2007a). In these cells, PrP^C^ was shown to accelerate the G1 to S phase transition in the cell cycle and to sustain proliferation by inducing the expression of Cyclin D1 through a PI3K/Akt pathway (Liang et al., 2007a). The PrP^C^-interacting protein(s) involved in this cascade remain(s), however, to be identified. Beyond proliferation, PrP^C^ overexpression in cancer cells was further shown to confer resistance to various cytotoxic agents (Mehrpour and Codogno, 2010) as well as invasive properties (Pan et al., 2006). For instance, PrP^C^ levels were shown to correlate with resistance to TNFα-induced cell death in the MCF-7 breast cancer cell line (Diarra-Mehrpour et al., 2004). Very recently, PrP^C^ was found to interact with the cell surface protein CD44 in adriamycin-resistant breast cancer cells, and to promote their proliferation and migration (Cheng et al., 2013). Interestingly, CD44 has been reported to be enriched at the cell surface of various types of tumor-initiating cells, which bear similarities with embryonic or adult stem cells and are often referred to as cancer stem cells (CSCs) (Medema, 2013). It is also noteworthy that CSCs have been associated with increased resistance to antitumor treatments (Singh and Settleman, 2010). In line with the above-mentioned role of PrP^C^ in the self-renewal of stem cells, Du et al. depicted a population of CD44^+^PrP^C+^ cells from primary colorectal tumors endowed with enhanced tumor-initiating and metastatic capacity (Du et al., 2013). At a mechanistic level, PrP^C^ was shown to promote an epithelial to mesenchymal transition (EMT) through the regulation of the Twist transcription factor (Du et al., 2013). These observations are in agreement with the notion that the emergence of CSCs and EMT are intimately connected (Singh and Settleman, 2010).

A still unresolved question concerns the molecular mechanisms sustaining the enhanced expression of PrP^C^ in cancer cells. PrP^C^ expression has been shown to be increased in response to hypoxia in gastric cancer cell lines (Liang et al., 2007b). Other PrP^C^-inducing signals include oxidative (Sauer et al., 1999) and endoplasmic-reticulum (Dery et al., 2013) stresses. Some deregulation of PrP^C^ function may also arise with aging. Indeed, PrP^C^ was recently shown to accumulate in lipid rafts in the mouse aging brain (Agostini et al., 2013). Whether this change in PrP^C^ distribution also occurs in other tissues with aging is worth considering, since it would potentially impact on the recruitment of downstream signaling cascades. As observed in the context of neurodegeneration (Hirsch et al., 2014), the subversion of PrP^C^ function may over-activate src kinases and further promote alterations in lipid raft-initiated signaling pathways, known to be detrimental in cancer (Patra, 2008). Such changes may in turn have consequences on the cell local environment, and, in the case of stem cells, deregulate the interactions with their niche. This scenario is considered as a potential cause of CSCs emergence (Rezza et al., 2014), and this may have particular relevance with respect to aging.

## Open questions and therapeutic prospects

Harnessing the self-renewal and differentiation potential of stem cells represents a major challenge for regenerative medicine. The recent accumulation of data regarding the involvement of PrP^C^ in stem cell biology warrants further studying the molecular and cellular mechanisms sustaining the contribution of this protein to the proliferation of stem cells, their maintenance in an undifferentiated state, their capacity to respond to fate determination inputs and to implement a given differentiation program. Achieving this task is complicated by the multiplicity of PrP^C^ isoforms and partners, which may fulfill promiscuous functions. That PrP^C^ is required for efficient tissue repair after injury is clearly indicated in the context of bone-marrow reconstitution (Zhang et al., 2006) or muscle regeneration (Stella et al., 2010), which suggest that the mobilization of PrP^C^-dependent cascades via appropriate ligands may provide a fruitful approach to enhance the regeneration of lesionned tissues. As a prerequisite, manipulating conditions would need to be carefully adjusted in order to control activating signals, given the pathological implications that may ensue from PrP^C^ over-activation.

Finally, the emerging roles of PrP^C^ in stemness on the one hand and in various aspects of cancer cell biology on the other hand bring new light on this already fascinating molecule. Given the relationship between stem cells and oncogenesis, advance in the understanding of the role played by PrP^C^ in stem cells is likely to illuminate the issue of its contribution to tumorigenesis and vice-versa. One major remaining challenge is to decipher the mechanisms controlling the expression levels of PrP^C^ in normal and cancer stem cells. While the cues underlying the induction of PrP^C^ during embryonic development are elusive, several cancer-associated conditions, including hypoxia (Liang et al., 2007b), oxidative (Sauer et al., 1999) or endoplasmic reticulum (Dery et al., 2013) stresses have been reported to activate PrP^C^ transcription. More broadly, increasing our knowledge of the regulation of PrP gene expression may help design novel strategies for therapeutic intervention in cancer, beyond directly targeting PrP^C^ through antisense oligonucleotides (Meslin et al., 2007) or monoclonal antibodies (Du et al., 2013).

To conclude, progress in the stem cell and cancer fields should increase our knowledge of how PrP^C^, as a cell surface receptor or co-receptor, connects cells with their environment to drive adaptive, homeostatic responses and how this function is corrupted in disease-associated states.

### Conflict of interest statement

The authors declare that the research was conducted in the absence of any commercial or financial relationships that could be construed as a potential conflict of interest.
